# Complete Genome Sequence of *Vibrio alginolyticus* ZJ-T

**DOI:** 10.1128/genomeA.00912-16

**Published:** 2016-09-01

**Authors:** Yiqin Deng, Chang Chen, Zhe Zhao, Xiaochun Huang, Yiying Yang, Xiongqi Ding

**Affiliations:** a Key Laboratory of Tropical Marine Bio-Resources and Ecology, South China Sea Institute of Oceanology, Chinese Academy of Sciences, Guangzhou, China; b Guangdong Provincial Key Laboratory of Applied Marine Biology, South China Sea Institute of Oceanology, Chinese Academy of Sciences, Guangzhou, China; c University of Chinese Academy of Sciences, Beijing, China; d Xisha/Nansha Ocean Observation and Research Station, South China Sea Institute of Oceanology, Chinese Academy of Sciences, Guangzhou, China

## Abstract

*Vibrio alginolyticus* is a ubiquitous Gram-negative bacterium which is normally distributed in the coastal and estuarine environments. It has been suggested to be an opportunistic pathogen to both marine animals and humans, Here, the completed genome sequence of *V. alginolyticus* ZJ-T was determined by Illumina high-throughput sequencing.

## GENOME ANNOUNCEMENT

*Vibrio alginolyticus* is a halophilic anaerobic Gram-negative bacterium that has drawn much attention recently worldwide (1). This organism can cause epidemic vibriosis in many commercial fish, leading to a high mortality and serious economic losses (2, 3). In addition, it is increasingly recognized as a potential threat to humans by causing food poisoning, intestinal inflammation, and wound infections (4, 5). A recent report showed that *V. alginolyticus* has now been listed as one of the most common pathogens, together with *Vibrio parahaemolyticus* and *Vibrio vulnificus*, in the United States (6).

*V. alginolyticus* ZJ-T is the translucent/smooth variant of ZJ-51, which was isolated from diseased *Epinephelus coioides* in Zhanjiang, Guangdong Province, China (7). *V. alginolyticus* ZJ-T was grown overnight in tryptic soy broth (TSB) (BD, USA). The overnight culture was inoculated into 20 ml of fresh TSB with an initial optical density at 600 nm (OD_600_) of 0.01 and cultured at 30°C with vigorous shaking for 8 h (OD_600_, 5.0). Two milliliters of culture was pelleted by centrifugation, and the bacterial cells were washed three times with 1× phosphate-buffered saline (PBS). DNA extraction was conducted with the QIAamp DNA minikit (Qiagen, Germany). Qualities and quantities were double checked by measuring the OD_260/280_ and OD_260/230_ with NanoDrop 2000 (Thermo, USA) and agarose gel electrophoresis. A concentration of 50 ng/µl was used for sequencing. The genome of *V. alginolyticus* ZJ-T was paired-end sequenced at the Beijing Genomics Institute (BGI) through Illumina high-throughput sequencing, with a genome coverage of 200×. The raw data were subjected to quality control (QC), and the resulting clean data were primarily assembled with SOAP*denovo* 1.05 and error corrected with SOAPaligner/soap2 (http://soap.genomics.org.cn/soapaligner.html). Finally, gaps were closed by PCR. The complete genome of ZJ-T was obtained, consisting of two circular chromosomes, with a total size of 5,406,094 bp and G+C content of 44.71%. Coding sequence prediction and annotation were conducted using the NCBI Prokaryotic Genome Annotation Pipeline (http://www.ncbi.nlm.nih.gov/genome/annotation_prok/). A total of 4,866 genes were predicted, including 4,664 coding genes, 46 pseudogenes, 28 rRNAs, 124 tRNAs, and four noncoding RNAs (ncRNAs). Additionally, tandem repeats (TRs), which were used as a marker for evolution of species because they are exclusively present in specific species and can be heritable, were analyzed using the Tandem Repeats Finder (TRF) (http://tandem.bu.edu/trf/trf.html). A total of 93 TRs, including 35 minisatellite DNA and 18 microsatellite DNA, have been predicted, with a total length of 46,419 bp, which possesses 0.86% of the whole-genome sequence. However, no clustered regularly interspaced short palindromic repeat (CRISPR) was found using CRISPRFinder (http://crispr.u-psud.fr/Server/CRISPRfinder.php).

Here, the complete genome sequence of *V. alginolyticus* ZJ-T was first elucidated. The data will facilitate future comprehensive comparisons and phylogenetic analyses of vibrios and provide genome information for the study of this opportunistic pathogen.

### Accession number(s).

The complete genome sequence of *V. alginolyticus* ZJ-T has been deposited at GenBank under the accession numbers CP016224 and CP016225.
